# Altered morphological dynamics of activated microglia after induction of *status epilepticus*

**DOI:** 10.1186/s12974-015-0421-6

**Published:** 2015-11-04

**Authors:** Elena Avignone, Marilyn Lepleux, Julie Angibaud, U. Valentin Nägerl

**Affiliations:** Interdisciplinary Institute for Neurosciences, CNRS UMR 5297, 33077 Bordeaux, France; Université de Bordeaux, CNRS UMR 5297, 33077 Bordeaux, France

**Keywords:** Microglia, Microglial dynamics, Epilepsy, Inflammation, Two-photon microscopy, Laser lesion

## Abstract

**Background:**

Microglia cells are the resident macrophages of the central nervous system and are considered its first line of defense. In the normal brain, their ramified processes are highly motile, constantly scanning the surrounding brain tissue and rapidly moving towards sites of acute injury or danger signals. These microglial dynamics are thought to be critical for brain homeostasis. Under pathological conditions, microglial cells undergo “activation,” which modifies many of their molecular and morphological properties. Investigations of the effects of activation on motility are limited and have given mixed results. In particular, little is known about how microglial motility is altered in epilepsy, which is characterized by a strong inflammatory reaction and microglial activation.

**Methods:**

We used a mouse model of *status epilepticus* induced by kainate injections and time-lapse two-photon microscopy to image GFP-labeled microglia in acute hippocampal brain slices. We studied how microglial activation affected the motility of microglial processes, including basal motility, and their responses to local triggering stimuli.

**Results:**

Our study reveals that microglial motility was largely preserved in kainate-treated animals, despite clear signs of microglial activation. In addition, whereas the velocities of microglial processes during basal scanning and towards a laser lesion were unaltered 48 h after *status epilepticus*, we observed an increase in the size of the territory scanned by single microglial processes during basal motility and an elevated directional velocity towards a pipette containing a purinergic agonist.

**Conclusions:**

Microglial activation differentially impacted the dynamic scanning behavior of microglia in response to specific acute noxious stimuli, which may be an important feature of the adaptive behavior of microglia during pathophysiological conditions.

**Electronic supplementary material:**

The online version of this article (doi:10.1186/s12974-015-0421-6) contains supplementary material, which is available to authorized users.

## Background

Microglia are the resident macrophages of the central nervous system (CNS), acting as its first line of defense to cordon off brain lesions, to phagocytize cellular debris, and to release signaling molecules critical for cell survival [1].

Under physiological conditions, microglia show a uniform distribution and occupy distinct spatial domains with minimal overlap [2]. A hallmark of microglia is their highly dynamic and motile nature, which allows them to react rapidly and intervene locally for effective maintenance and control of brain homeostasis. Indeed, they continuously scan the parenchyma of the brain in an apparently random fashion (called basal motility), rapidly extending and retracting their highly branched processes [3–6] and contacting practically all other cell types and neuronal compartments, including synapses [7, 8].

In response to an acute lesion, microglia rapidly project their processes towards sites of danger signals (called directional motility; [3, 4, 9]), presumably to probe and contain the damage and to protect the surrounding cells [10].

Due to their role as immune-competent cells, a variety of signals are expected to attract microglial processes. In particular, they are attracted by adenosine triphosphate (ATP) and its derivatives. Indeed, activation of purinergic receptors plays a major role for microglial dynamics [3, 11]. In particular, extracellular ATP affects basal motility [3, 12], while activation of purinergic P2Y12 receptors (P2Y12R) mediates directional motility induced by laser lesions [13, 14].

Under pathological conditions, microglia change many of their morphological and molecular properties through a series of long-term transformations, called microglial activation [15, 16]. Microglial activation plays a critical role in the inflammatory reaction associated with a variety of diseases, and it is a prominent feature in the brain following *status epilepticus* (SE). SE is a seizure lasting several minutes and that may occur in patients with a history of epilepsy or as a consequence of a variety of insults, such as trauma, febrile seizure, and stroke. The inflammatory reaction induced by SE may contribute to the progression towards recurrent (chronic) epilepsy and to common associated neuropsychiatric comorbidities such as depression, memory impairment, and autism spectrum disorders [17]. Microglial activation, which plays a central role in orchestrating the inflammatory reaction, is accompanied by morphological and functional changes that may influence their motility and could thus compromise their housekeeping capacities. In the context of SE, it is unclear how microglial activation affects the motility of microglia and their ability to respond to noxious signals. A down- or up-regulation of microglial motility is conceivable as microglia are known to retract their processes and to exhibit boosted purinergic responses at the same time after SE [18–20].

To explore this question, we performed time-lapse two-photon imaging of microglia in acute hippocampal brain slices two days after induction of SE in mice. We assessed the impact of microglial activation on basal and directional microglial motility in response to focal laser lesions as well as to local application of an ATP analog.

## Methods

### Animals and SE model

All experiments followed Inserm and European Union and institutional guidelines for the care and use of laboratory animals (Council directive 2010/63/EU) and have been validated by the local ethics committee of Bordeaux (n° A50120200). The heterozygous CX3CR1^+/eGFP^ mice used in this study were obtained by crossing CX3CR1^eGFP/eGFP^ with C57BL/6 (Janvier, Le Genest Saint Isle, France) wild-type mice. To avoid non-specific activation of microglia, animals were kept in an animal facility free of specific pathogenic organisms until the experiments.

To induce SE, 30- to 40-day-old male mice received two intraperitoneal (i.p.) kainate (KA) injections (15 and 5 mg/kg) at an interval of 30 min. Littermate male mice injected with PBS were used as control. The KA injections induced crises scored according to the Racine scale of level 3 (rear into a sitting position with forepaws shaking) to 5 (continuous rearing and falling). Mice, which did not reach level 3 after 1 h received a third injection (5 mg/kg). The multiple injection protocol provided a better control of the crisis and reduced mortality (less than 10 %).

The overall crisis of each animal was evaluated according to a modified Racine scale, which also considered the duration of the crisis. The crisis was classified as mild (level 2 of Racine’s scale: rigid posture with straight and rigid tail), intermediate (level 3 of Racine’s scale, duration shorter than 2 h), intense (level 3 of Racine’s scale with episode 4 or 5, duration shorter than 3 h), or severe (erratic behavior such as jumping or walking backwards or crisis duration longer than 3 h).

### Preparation of acute brain slices

Hippocampal slices were prepared 48 h after i.p. injections. Mice were sacrificed by cervical dislocation. The brain was then quickly removed and placed in ice-cold artificial cerebrospinal fluid (aCSF) saturated with carbogen (95 % O_2_/5 % CO_2_) and in which NaCl was replaced by sucrose (in mM: 210 sucrose, 2 KCl, 26 NaHCO_3_, 1.25 NaH_2_PO_4_, 10 glucose, 0.2 CaCl_2_, 6 MgCl_2_; pH 7.4, osmolarity 310 mOsm). Transverse 350-μm-thick slices were cut using a vibratome (VT1200, Leica, Mannheim, Germany), transferred to a heated (33 °C) holding chamber containing carbogenated (95 % O_2_/5 % CO_2_) standard aCSF (in mM: 124 NaCl, 3 KCl, 26 NaHCO_3_, 1.25 NaH_2_PO_4_, 10 glucose, 2 CaCl_2_, 1 MgCl_2_, pH 7.4, osmolarity 305 mOsm/L) for 45 min, and then maintained until the experiment at room temperature for a maximum of 3 h.

### Time-lapse two-photon imaging

Individual slices were transferred to a submerged recording chamber and continuously perfused with carbogenated aCSF (3 ml/min) at 33 °C. Images were acquired with a 40X water immersion objective (NA 1.0), using a commercial upright two-photon laser-scanning fluorescence microscope (Ultima, Prairie Technologies, Middleton, Wisconsin, USA and Axio Examiner, Zeiss, Oberkochen, Germany). For two-photon excitation, a Ti:Sapphire laser (Mai Tai, Spectra-Physics, Darmstadt, Germany) was tuned to 900 nm. Four-dimensional image stacks (*x*, *y*, *z*, *t*) were acquired in the *strata radiatum*/*lacunosum-moleculare* in the CA1 area of the hippocampus. The voxel size was 200 × 200 × 1000 nm^3^ in all experiments measuring basal motility and responses to laser lesion, while it was 300 × 300 × 1000 nm^3^ in most of the experiments with the ATP analog. The *z*-stacks were 11 to 25 μm thick and acquired at intervals between 25 and 60 s. We did not detect any signs of photo-induced damage during extended time-lapse acquisitions lasting up to 1 h.

To induce a small laser lesion, a region of 1.6 × 1.6 μm was repeatedly illuminated for 20–50 times with an elevated intensity of the two-photon laser, which reliably induced a microglial response.

A puff of the P2Y12R agonist 2-Methylthioadenosine diphosphate trisodium salt (2Me-ADP, 100 μM) was applied by manual pressure through a glass pipette gently inserted in the slice to limit mechanical damage. No microglial responses were observed when a pipette containing aCSF was inserted and a similar pressure was applied.

To reduce effects from the slicing procedure, we imaged microglia that were fully embedded in the brain slice, at least 50 μm below the surface of the slice, which is a depth where neurons are usually intact [21]. At this imaging depth, microglial morphology was comparable to what has been observed in perfusion-fixed animals (data not shown) and in vivo [3, 4]. Furthermore, in our experimental conditions, basal motility was similar to what has been reported for cortex and spinal cord in vivo [3, 4, 9].

### Image analysis

Analysis was carried out with ImageJ (National Institute of Health). Analysis of microglial cell body size was performed on maximal intensity projection images, considering only the cells having the cell soma fully contained within the 3D image stack. Image analysis was done blindly with respect to crisis level. In order to avoid subjective interpretation of the crisis level, only scores by the same person were considered for the correlation between crisis level and microglial morphology. Cell bodies were measured using the “magic wand” tool in ImageJ on the maximal intensity projection image. When objects from a different focal plane contaminated the measurement, a subset of images containing only the cell body was used for the projection. The number of primary processes was assessed in 3D stacks. The longest process was verified in the 3D stack, then drawn by hand and measured in the 2D projection. Images of microglia were taken in two to three hippocampal slices per animal. Morphological parameters were measured in all images, and the median was taken as a representative value of each animal. To assess the relationship between cell body size and dynamics, the median of cell body measurements per slice in each experiment was taken as representative value.

To compensate for *x*-*y* movements, images of the temporal series were aligned using the following ImageJ plug-ins: StackReg, MultistackReg, and PoorMan3Dreg [22]. To analyze the movement of single processes, the plug-in MtrackJ [23] and custom written programs in MATLAB (MathWorks, France) were used. Each movement was classified as elongation, retraction, or stationary, according to its velocity relative to a fixed reference point (lesion site or pipette tip for directional motility). In the case of basal motility, the reference point was defined by where the process emerged from the parent process. The territory explored was calculated as maximum-minimum distance from the point of reference.

To estimate the overall velocity at which the fluorescent processes elongated towards the target (pipette or laser lesion site), a series of concentric circles were drawn as a region of interest and the fluorescence was measured in concentric rings (Additional file 1: Figure S1). The velocity was defined as the distance between two rings divided by the time between their maxima of the fluorescence signal. We considered only the experiments in which we could clearly identify at least three peaks of fluorescence in three coronal sections, thus two velocities, and the mean was taken as the representative velocity of each experiment (Additional file 1: Figure S1).

The territory explored by a single process was calculated as the linear distance between the most distant and closest points reached by a single process with respect to its parent branch, irrespective of elongation or retraction. For each slice, 7–10 processes from several cells were analyzed.

### Statistics

Data values are presented as mean ± SEM or median with quartile. Statistical significance was established with statistical tools in MATLAB or Origin (OriginLab, RITME Informatique, France). First, we tested whether data were sampled from populations that follow Gaussian distributions using the method of Kolmogorov and Smirnov. For sample populations with non-Gaussian and Gaussian distributions, a non-parametric (Mann-Whitney) or parametric tests (*t* test with Welch’s correction) were used to assess differences in the median (or mean). In case of paired data, the non-parametric Wilcoxon signed-rank test was used. The Kolmogorov and Smirnov test was used to compare distributions of pooled values across all experiments between two groups of animals. Statistical significance was established at *p* < 0.05 and *p* < 0.01. *N* represents the number of animals, while *n* represents the number of cells, experiments, or processes as indicated in the text.

## Results

### Microglial activation after status epilepticus

**Fig. 1:**
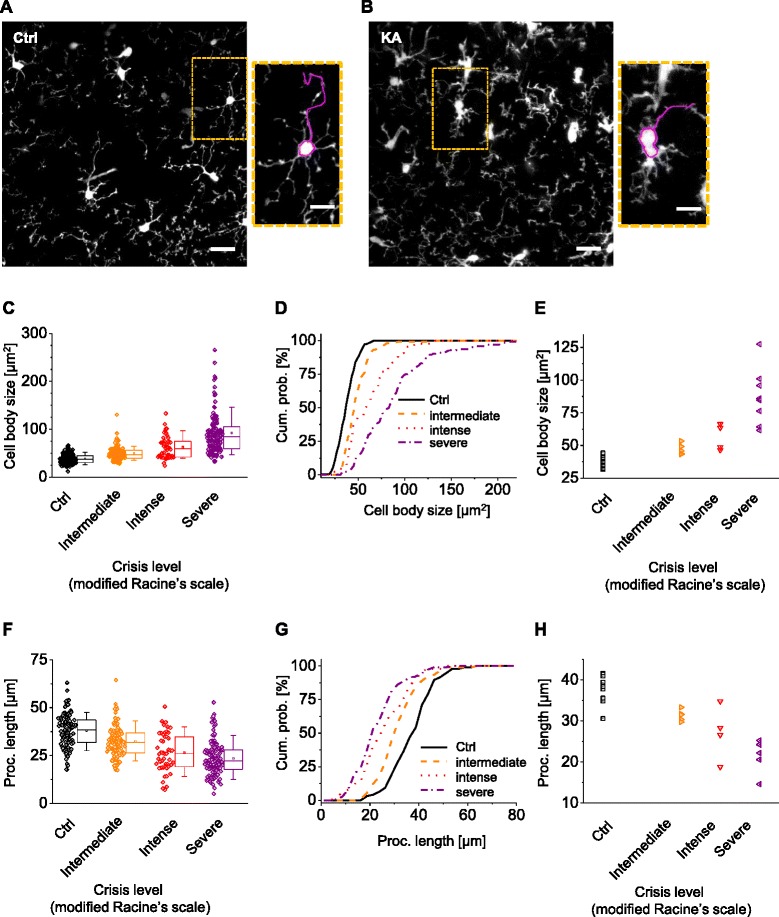
Cell body size correlates with the severity of *status epilepticus*. **a, b** Maximal intensity projection (MIP, *z* = 19 μm) images of microglial cells obtained in control mice (**a**) and in mice two days after kainate i.p. injection (**b**). The insets show higher magnification images. The *line around cell bodies* was drawn in a semi-automatic way to measure cell body size. The *line on the process* was based on the 3D image stack to avoid projection artifacts. Note the increase in cell body size of microglia and decrease of the longest process after SE. Scale bars 10 and 6 μm in the insets. **c** Soma size measurements obtained in all experiments classified according to the crisis level of animals, their median, and quartile value. **d** Cumulative probability of cell body size grouped according to the crisis level of animals, scored according to modified Racine’s scale. Note the progressive shift to the right with the increase of the severity of the induced SE. **e** Relationship between the median of cell body size for each animal and its crisis level or in control (*ctrl*, *black*). **f** Measurements of longest processes obtained in all experiments classified according to crisis level of animals, their median, and quartile value. **g** Cumulative probability of data represented in (**f**) grouped according to crisis level. Note the progressive shift to the left with increasing crisis severity. **h** Relationship between the median of the longest processes for each animal and its crisis level or in control (*ctrl*, *black*)

We imaged microglia in acute hippocampal brain slices from Cx3Cr1^+/eGFP^ mice by two-photon microscopy and analyzed their morphological dynamics. As previously described [18, 21, 24], 48 h after injection, we observed an increase in the number of microglia in KA-treated animals, indicative of microglial activation. Furthermore, in KA-treated animals, microglial cell bodies were substantially larger and processes were shorter compared to control animals (Fig. 1a, b). We checked whether the degree of morphological changes after SE induction correlated with the severity of SE, as classified according to a modified Racine scale (see Methods for details). While no significant differences in cell body sizes were observed between control (*n* = 163, *N* = 10) and animals with mild seizures (*n* = 21, *N* = 2, KS test, *p* = 0.29), the cumulative probability plot of the distribution obtained from animals with intermediate (*n* = 106, *N* = 5), intense (*n* = 43, *N* = 4), and severe seizures (*n* = 126, *N* = 8, KS test, *p* < 0.01, compared to previous level; Fig. 1c, d) was progressively shifted to the right. Moreover, in experiments where the animals exhibited intense and protracted seizures, microglia took on an amoeboid shape and migrated to the pyramidal cell body layer (Additional file 2: Figure S2). Microglial cell shape varied widely in KA-treated animals, even within the same slice. The variation was larger for severe crises, where we could observe hyper-ramified cells, as well as amoeboid-like shapes. We analyzed two morphological parameters: the number of primary processes and the longest process identifiable in each cell. The number of primary process was not statistically different between the control (median 6, *n* = 85) and animal with intermediate seizure (median 5.5, *n* = 56; KS test 0.99), while it was different between control animals and animals with severe seizure (median 7, *n* = 36; KS test 0.028). Similar to cell body size, we observed a progressive behavior, with the process length shifting towards smaller values for more intense crises (Fig. 1f, h). The median values were 38.5, 31.8, 26, and 22 μm for control (*n* = 96, *N* = 10), intermediate (*n* = 91, *N* = 5), intense (*n* = 49, *N* = 4), and severe (*n* = 115, *N* = 8) crisis levels, respectively. All distributions were significantly different from the respective previous level (KS < 0.01), except between the severe and intense crisis (KS = 0.08). These results are in general agreement with changes reported in the literature in different animal models [24–27].

Taken together, these results show that there is a strong correlation between seizure severity and morphological changes (Fig. 1e–h), indicating that cell body size can be used as a reliable proxy of seizure intensity and microglial activation.

### Effect of microglial activation on basal motility of microglial processes

To establish whether and how microglial activation induced by SE affects the ability of microglia to patrol brain parenchyma, we tracked the morphological dynamics of microglial processes by time-lapse two-photon imaging in acute hippocampal slices.

**Fig. 2:**
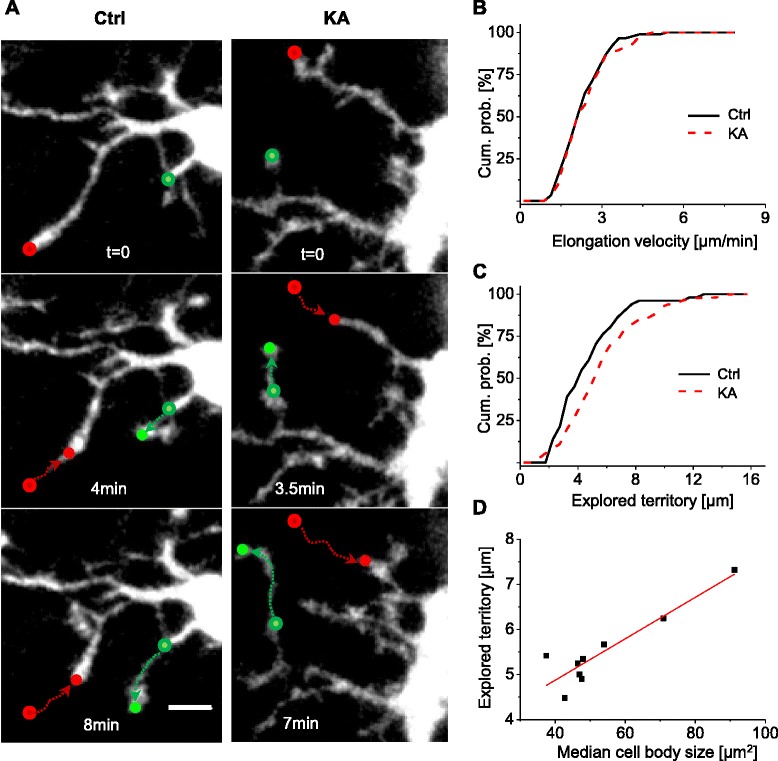
Activated microglia scan larger territory, without changing their velocity. **a** Maximal intensity projection of time-lapse two-photon images at different time points during spontaneous movements of microglial processes in control (*left column*) and 48 h after induction of SE (*right column*). The figure shows retracting (*red symbols*) and elongating processes (*green symbols*), with the starting/arriving points marked by *circles*. Scale bar, 5 μm. **b** Cumulative probability of all elongation movements by single process tips measured in control (*black*) and KA-injected *(red) * animals shows no difference between the two groups (*p* = 0.99, KS test). **c** Cumulative probability of territory explored by all single process tips measured in control (*black*) and KA-injected *(red) * animals. *p* = 0.019, KS test. **d** Relationship between the median of explored territory by microglial processes versus the median of the cell body size in each experiment. The *red line* represents the linear fit (*r* = 0.8)

First, we analyzed the motility of microglial processes under baseline conditions in control and KA-treated animals (Fig. 2). To this end, we measured the average velocity of the tips of individual microglial processes based on the maximal intensity projections of 3D image stacks acquired every 45 s for up to 8 min.

The average tip velocity was indistinguishable between KA-treated and control tissue (ctrl: 2.38 ± 0.08 μm/min, *N* = 7; KA: 2.46 ± 0.10 μm/min *N* = 6; *p* = 0.58, *t* test). Similarly, no difference was found between the velocity distributions of all processes for the two groups (Fig. 2b; ctrl: *n* = 89; KA: *n* = 71; KS = 0.99), despite the significant difference in their average cell body size (ctrl: 42.2 ± 2.5 μm^2^; KA: 73.5 ± 9.1 μm^2^; *p* = 0.004), confirming that KA injections indeed had induced microglial activation.

Performing extended time-lapse imaging experiments (acquiring 3D image stacks every 30 s for up to 30 min), we also analyzed the speed at which individual microglial processes elongated and retracted, which is a more refined measure of microglial process motility. Comparing elongation and retraction velocity in the same process did not turn up any significant differences, neither in control (*p* = 0.46; Wilcoxon signed-rank test; *n* = 51 processes; obtained in experiments in three animals) nor KA-treated animals (*p* = 0.95; Wilcoxon signed-rank test; *n* = 136 processes; obtained in nine experiments in five animals; data not shown).

Finally, we determined the size of the territory explored by microglial processes as the maximal linear covered distance (see Methods for details). The size of the territory was highly variable (ranging from 1 to 15 μm). Yet, on the whole, microglial processes explored a significantly larger territory in KA-treated than in control animals (median ctrl: 4.18 μm; *n* = 51 processes; *n* = 3 experiments; three animals; median KA: 5.38 μm; *n* = 152 processes; *n* = 9 experiments; five animals; *p* = 0.019; Fig. 2c). Moreover, there was a strong positive correlation between microglial cell body size (median value per animal) and the average distance covered by microglial processes in KA-treated animals (*r* = 0.8; Fig. 2d).

Taken together, our analysis indicates that basal motility of microglia was preserved after microglial activation. Microglial processes moved at the same speed but covered a larger distance in KA-treated animals as compared with control animals.

### Directional motility towards a pipette containing P2Y12R agonist

**Fig. 3:**
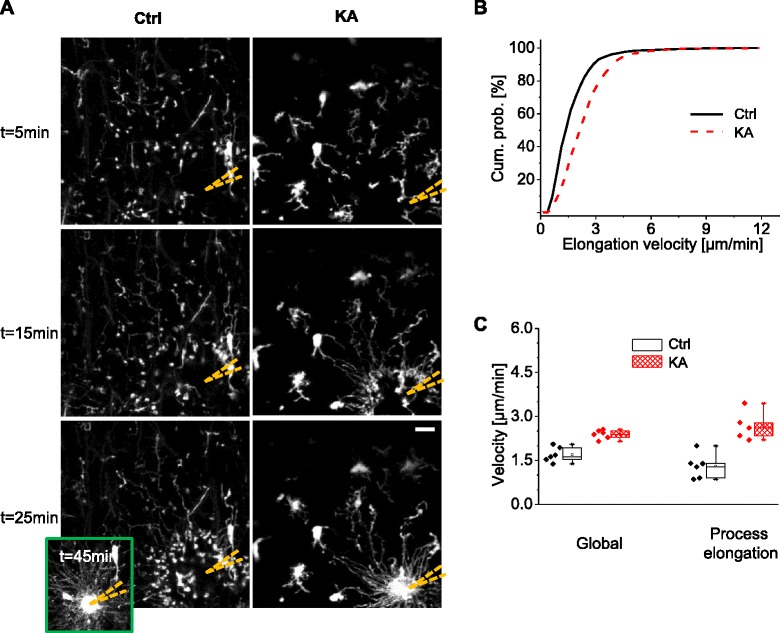
Faster process motility of activated microglia towards a pipette containing 2Me-ADP. **a** Maximal intensity projection of two-photon images at different time points after the insertion (at *t* = 0) of a pipette containing 2Me-ADP (100 μM) in slice from control (*left column*) or kainate-treated (*right column*) animals. At *t* = 25 min (*right column*), the processes of activated microglia have reached the pipette (*bottom images*), whereas those of microglia from control mice reached their target only after 45 min (*inset picture*). Scale bar, 15 μm. **b** Cumulative probability of all elongation movements by single process tips measured in control (*black*) and KA-injected (*red*) animals (*p* < 0.001, KS test). **c** Velocity measured with two different methods in control (*black*) and KA-injected (*red*) animals. Global velocity was assessed measuring the fluorescence in concentric rings around the pipette tip. Process elongation velocity was evaluated considering the median of average elongation tip velocity of several processes in the experiment. Both methods revealed a higher velocity in KA-injected animals compared to control (*p* < 0.01, *t* test)

We next assessed the ability of activated microglia to rapidly react to potentially noxious signals. Consistently with previous reports, microglial processes were attracted by ATP analog 2Me-ADP (Additional file 3; Additional file 4), an agonist of P2Y12R, while cell bodies did not move during the observation time (up to 1 h) [3, 13, 18, 28]. We tracked individual microglial processes during their movements towards a patch pipette containing the P2Y12R agonist. We observed that it took microglial processes about half the time to reach the pipette tip in KA-treated animals compared to control experiments (ctrl: 58.2 ± 5.7 min, *n* = 6, *N* = 4; KA: 29.0 ± 3.7 min, *n* = 6, *N* = 4; *p* < 0.01, *t* test; Fig. 3a), confirming a previous study based on wide-field imaging in acute brain slices [18].

In two KA experiments, microglial processes failed to be attracted by the 2Me-ADP-containing pipette. In both cases, slices were obtained from mice, which had undergone particularly strong crisis and whose microglia showed signs of extreme activation. Indeed, their cell bodies were very large and fragmented and mostly devoid of processes (Additional file 2: Figure S2), suggestive of strong phagocytic activity. This data set was excluded from the statistical analysis of the morphological dynamics.

To analyze the directional motility in greater details, we compared the distributions of all elongation steps made by single microglial processes. The distribution observed in KA-injected animals was substantially shifted to the right compared to the control case, indicating increased velocity (ctrl: median 1.49 μm/s, *n* = 1450 movements; KA: median 2.25 μm/s, *n* = 740 movements; KS test, *p* < 0.001; Fig. 3b). We then calculated the mean velocity of several single processes in each experiment (Fig. 3c), which confirmed the difference between the two experimental groups (*t* test, *p* < 0.01). Similar results were obtained by measuring the fluorescence of microglial processes passing through concentric rings drawn around the pipette (global velocity, see the “Methods” section for details). The mean velocity was 1.30 ± 0.17 μm/min (*n* = 6) for the control group and 2.68 ± 0.22 μm/min (*n* = 5) for the KA group (*p* < 0.01, *t* test; Fig. 3c).

The analysis of single processes revealed that some of them abruptly stopped and retracted and/or started to scan the territory in an apparent random fashion, even though initially they appeared to move towards the pipette tip. Therefore, we compared the retraction velocity between KA-treated and control animals. In contrast to elongation velocity, the distributions of retracting velocity were not statistically different (ctrl: median 1.38 μm/min, *n* = 97; KA median 1.73 μm/min, *n* = 57; *p* = 0.18, KS test). Consistently, elongation was more rapid than retraction in the KA group (*p* < 0.001, KS test), while in the control group elongation and retraction were not statistically different (*p* = 0.19, KS test; data not shown).

Taken together, our analysis shows that microglial activation affected the motility of microglial processes towards a pipette containing a purinergic agonist by specifically boosting the elongation speed, while the retraction speed was unaltered.

### Directional motility towards a laser lesion

**Fig. 4:**
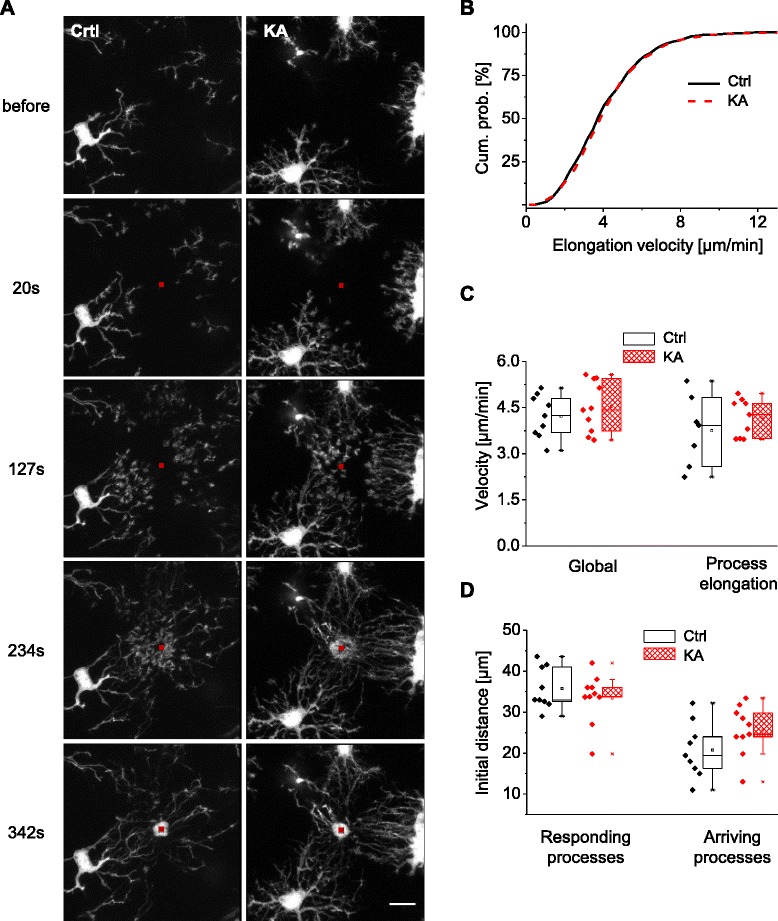
Microglial activation does not affect process motility towards a laser-induced lesion. **a** Examples of maximal intensity projection of two-photon images at different time points after the induction of lesion with a laser in a small portion of the slice (*red square*) in control (*left column*) and in KA-injected (*right column*) animal. Scale bar, 10 μm. **b** Cumulative probability of all elongation movements by single process tip measured in control (*black*) and KA-injected (*red*) animals. *p* = 0.17, KS test. **c** Global velocity and process elongation velocity measured in control (*black*) and KA-injected animals (*red*). None of the two methods shows a statistically significant difference between the two groups (*p* = 0.38 and 0.40 for global and process elongation, respectively, *t* test). **d** Evaluation of the area of influence of the laser lesion in control (*black*) and KA-injected (*red*) animals. Each *dot* represents the most distant process that still reacted to the lesion (*responding processes*) or actually arrived at the lesion site (*arriving processes*). None of the two groups showed a statistically significant difference (*p* = 0.39 and *p* = 0.16 for responding and arriving, respectively, *t* test)

In order to assess how microglial activation affects the ability of microglia to direct their processes towards sites of acute physical injury in brain tissue, we induced a laser lesion in a small region (1.6 × 1.6 μm) and measured the velocity of microglial processes moving towards the lesion site. Laser lesions have been used before in in vivo [3, 4] and acute slice studies [10]. In agreement with previous reports, we observed that many microglial processes rapidly switched from a basal scanning mode to directional motility towards the lesion site (Fig. 4a, Additional file 5; Additional file 6). In many instances, newly formed processes grew out from the cell body and from existing processes and moved towards the lesion site. Similar to the case of the ATP analog, cell bodies did not move during observation time (up to 30 min). The number of processes involved in the response was highly variable between experiments, depending on the distance to the lesion site and presumably also on the type of the affected region (e.g., neuronal dendrites or soma, astrocyte, blood vessel).

Microglial processes typically reached the lesion site within a few minutes (examples in Fig. 4a, Additional file 5; Additional file 6) for both experimental groups (ctrl: median values 4.23 min; *n* = 9, *N* = 6; KA: 4.48 min; *n* = 11, *N* = 6; *p* = 0.17; Mann-Whitney test), even though their median cell body sizes were substantially different (ctrl: 38.6 ± 2.64; KA: 64.7 ± 5.8; *p* = 0.001).

We then analyzed the global and elongation velocity of each process induced by the laser lesion. The elongations made by all analyzed processes did not show any statistically significant differences between the experimental groups (ctrl: median 3.84 μm/s; *n* = 817 movements, *N* = 6; KA: median 3.99; *n* = 1231 movements, *N* = 6; *p* = 0.4; KS test; Fig. 4b). Similarly, no statistically significant difference was found in the elongation velocity measured in each experiment (assessed as a mean of single processes) (ctrl: 4.22 ± 0.23 μm/min, *n* = 9, *N* = 6; KA: 4.53 ± 0.26 μm/min; *n* = 10, *N* = 6, *p* = 0.40, *t* test; Fig. 4c). Likewise, no differences were observed for global velocity between the experimental groups (ctrl: 3.75 ± 0.43 μm/min, *n* = 7; KA: 4.16 ± 0.2 μm/min, *n* = 9; *p* = 0.38, *t* test; Fig. 4c).

In summary, unlike the elevated directional motility towards a 2Me-ADP-containing pipette, the velocity towards a laser lesion was not measurably affected by microglial activation.

### Spatial extent of microglial responses to 2Me-ADP and laser lesions

In our experimental conditions, the velocity of the directional motility induced by laser lesions was roughly three times higher than the velocity induced by the P2Y12R agonist. However, the time it took to completely surround the pipette tip was almost 15 times longer than the time to surround the lesion site. To better understand this apparent discrepancy, we characterized in more detail the effects of 2Me-ADP application and laser lesions on directional motility.

We determined the spatial extent of the microglial reaction, i.e., the area over which microglial processes responded to a locally applied trigger stimulus.

Microglial processes were attracted over large distances in response to 2Me-ADP (ctrl: >100 μm, Additional file 3). By comparison, the distance was much smaller for laser lesions (laser maximal distance, ctrl: 35.8 ± 1.7 μm, *n* = 9, Fig. 4d). Interestingly, not all processes that initially responded (responding process) actually reached the target area (arriving process), both for 2Me-ADP application and laser lesion (maximal distance arriving process, ctrl: 20.8 ± 2.4 μm, *n* = 9, Fig. 4d). The maximal distance of arriving or responding processes was not significantly different in KA-treated animals (*p* = 0.38 for responding, *p* = 0.16 for arriving), suggesting that despite the dramatic changes in morphology, the response zone of microglia to a laser lesion was not affected.

Interestingly, the responses to 2Me-ADP application frequently seemed to be coordinated among processes from different microglia. We observed that processes, which were closer to the pipette, started to move only after the more distal ones had caught up with them (Additional file 3; Additional file 4; Additional file 7: Figure S3), both in the control and KA groups. By comparison, this striking effect of coordination across many microglia cells in response to the ATP analog was much less obvious in the response to laser lesions, where microglial processes rushed to the lesion site more precipitously (Additional file 8: Figure S4, Additional file 5; Additional file 6).

Thus, 2Me-ADP application and laser lesions both effectively triggered microglial directional motility. However, the responses to these two triggers differed with respect to their sensitivity to/modulation by SE, their velocity, and the spatial extent of their responses as well as the level of coordination in their collective behavior, which resulted in large differences in the time to reach the target.

## Discussion

Microglia play a critical role in the health and homeostasis of the CNS. One of their main responsibilities is to avert impending threats by continuously scanning brain parenchyma and removing noxious substances and cellular debris that would otherwise accumulate and disrupt brain physiology. This ability is particularly important during neurodegenerative diseases when neural cell death and damage are rampant. Thus, it is important to understand how microglial motility is affected by pathological states. Using time-lapse two-photon imaging in acute brain slices, we examined in detail the ability of microglia to scan brain tissue and to respond to acute danger signals in the context of an animal model of SE. We observed that the basal velocity of microglial processes and their directional motility towards a laser lesion were unaffected, while the size of the territory scanned by individual microglial processes and their velocity towards a source of ATP analogs were markedly increased after the induction of SE. Our experiments indicate that microglial motility was not compromised by the activation of microglia caused by the induction of SE. Instead, microglial activation resulted in an enhanced scanning behavior, which may represent a state of heightened vigilance.

The mechanisms underlying microglial motility are not well understood yet. However, purinergic signaling seems to play a major role [6, 11]. The ATPase apyrase and the purinergic antagonist suramin both slow down baseline motility, while ATP increases it [3, 12]. Directional motility towards laser lesion strongly depends on P2Y12R, since it is almost abolished in P2Y12R knockout animals [13, 14]. Forty-eight hours after SE, P2Y12R are up-regulated [18], which would be expected to lead to an increase in directional motility. Here, we confirmed that the velocity towards a pipette containing the P2Y12R agonist is indeed increased. Surprisingly, we did not find any difference in directional motility between KA-injected and control animals in response to laser lesions. However, the two types of directional motility could be mediated by different mechanisms. While the P2Y12R agonist activates a specific signaling pathway, laser lesions are a blunter trigger, which is likely to engage multiple signaling pathways that define the microglial response. Microglial dynamics depend on actin polymerization [10]. In other cell types, motility and actin polymerization are largely controlled by small GTPases [29–31], which can be differently modulated by the activation of several receptors, including purinergic receptors [32, 33]. Thus, stimulation of different microglial membrane receptors may induce distinct forms of directional motility. Consistently, the spatial extent and collective behavior of microglial processes were quite distinct for the two types of induced directional motility. Furthermore, the velocity of the processes was higher for laser lesions than in response to the ATP analog, and it might have been already at the maximal speed the cell could sustain. Indeed, as far as we know, no increase in the velocity of laser-induced directional motility has been reported following any type of treatment.

The relationship between activation and motility has been investigated before in several mouse models of neurological disorders, showing highly heterogeneous results. In amyotrophic lateral sclerosis (ALS) at the preclinical stage, basal motility was reported to be similar to control, while the response to laser lesion involved more microglial processes, which moved with unaltered velocity [34]. However, at the clinical stage, amoeboid and activated microglia reportedly showed reduced injury-directed response as well as reduced basal motility [34]. In contrast, in an Alzheimer’s model, microglial processes moved faster towards an ATP-containing pipette [35], while responses to a laser lesion and phagocytosis were impaired [36], and basal motility remained unaffected [37]. A low dose of intraperitoneal injection of lipopolysaccharide (LPS), which activates microglia, showed no effects after two days on basal motility in vivo [38], although a decrease was observed 2 h after injection in acute slices [39]. However, higher LPS doses increased basal motility without affecting the time to reach the site of the laser lesion [40]. Interestingly, the relationship between activation and actin dynamics may be bi-directional, since impairment of actin dynamics shapes microglial functions [41].

Thus, the effects of microglia activation on motility cannot be easily generalized, because they unfold dynamically over time and depend strongly on the disease model. Furthermore, the type of motility must be taken into account when comparing different models, because they may be differently affected by microglial activation as our experiments demonstrate. We speculate that differential expression of P2Y12R may partially account for the variety in observations, as it seems to vary with activation status and mode of activation. On the one hand, enhanced P2Y12R expression is observed in the spinal cord three days after partial sciatic nerve ligation [42], as well as in human microglia under pathological conditions that promote alternative activation (M2; IL4- and IL-13, [43]). On the other hand, in post-mortem samples from the cerebral cortex of patients with multiple sclerosis (MS), P2Y12R expression is absent in microglia within the lesion zone [44]. Consistently, it gradually decreases in ALS and MS animal models [45]. Similar effects are observed when microglia activation is induced by LPS [13]. Thus, high activation characterized by an amoeboid shape may diminish P2Y12R expression. The lack of response in this study to a P2Y12R agonist in slices obtained from two animals, which had undergone a particularly long and severe crisis, is consistent with this hypothesis.

In addition to the velocity of microglial processes, it is important to consider the territory they explore, because it may influence their ability to interact with each other. Under physiological conditions, microglial processes hardly ever come into physical contact with each other. The avoidance behavior is abandoned when they detect danger signals and act together to isolate the source of the putative problem, implying that their mutual repulsion is dynamically regulated.

After SE, the spatial distribution of microglia was altered. Besides the twofold increase in the number of microglia [18], several cell bodies and cellular processes were found in the immediate proximity of each other (Additional file 9: Figure S5), which may reflect an alteration of the rules governing their physical interactions. Furthermore, the territory spanned by single processes was larger in KA-treated than control animals, which might reflect a compensation for shorter processes, allowing an efficient scanning of the territory. These phenomena could be due to the reduction of the signaling molecule(s) used for mediating the repulsive interactions.

## Conclusions

This study shows that, in contrast to other models of microglial activation, the state of activation induced 48 h after induction of SE does not hamper the sentinel role of microglia. Indeed, they can still patrol the environment, and they can react to stimuli, possibly even in a more efficient way. Furthermore, our study reveals that microglia exhibit a variety of motility behaviors, which are differentially affected by microglial activation, indicating that the dynamic behavior of microglia depends on their state of activation as well as the nature of the stimulus, which triggers the motility.

## Additional files

Additional file 1: Figure S1. Method to assess global velocity of microglial processes. **A** Concentric rings are drawn around the area of interest (laser lesion spot or 2Me-ADP-containing pipette) in the maximal intensity projection (MIP) image. **B** Global velocity is calculated by observing the fluorescent wave passing through the rings in the MIP video. Each curve represents the evolution of the normalized fluorescence in time in the corresponding ring (matched color in A). Additional file 2: Figure S2. Example of hyper-activated microglial cells in a slice obtained from a mouse with a long and severe crisis. Maximal intensity projection of two-photon images of the CA1 region in a hippocampal slice obtained from a KA-injected animal, which showed a particularly severe crisis. Microglia almost completely retracted their processes and accumulated around the *stratum pyramidale*, indicated by the dotted line. Scale bar, 30 μm. Additional file 3:
**Microglial response to P2Y12R agonist in control animal.** This movie shows the microglia response to a pipette containing 2Me-ADP in control animal. Processes distant as far as 100 μm responded to the stimulus. The processes coordinate their response to form a circle and arrive at the ATP analog source at the same time. New processes of microglia closer to stimulus start to be formed but started to move only after the more distal ones had caught up with them. Time expressed in minutes. Images are maximal intensity projection over a stack of 20 μm. Scale bar, 10 μm. Pixel sizes, 500, 300, and 200 nm. Additional file 4:
**Microglial response to P2Y12R agonist in KA-injected animal.** This movie shows the microglia response to a pipette containing 2Me-ADP in animal injected with KA. Comparing to control, processes reach the target in about half time. Time expressed in minutes. Images are maximal intensity projection over a stack of 20 μm. Scale bar, 10 μm. Pixel size, 250 nm. Additional file 5:
**Microglial response to laser ablation in control animal.** This movie shows the microglia movement before and after a small (1.6 × 1.6 μm) laser lesion (red spot) in control animal. Newly formed processes grew out from the cell body and from existing processes and moved towards the lesion site. Images are maximal intensity projection over a stack of 10 μm. Time expressed in minutes and seconds. Scale bar, 10 μm. Pixel size, 200 nm. Additional file 6:
**Microglial response to laser ablation in KA-injected animal.** This movie shows the microglia response to a small (1.6 × 1.6 μm) laser lesion (red spot) in KA-injected animal. In this movie, we can see that the response is not completely synchronized and processes do not approach the lesion site as a circle. Images are maximal intensity projection over a stack of 10 μm. Time expressed in minutes and seconds. Scale bar, 10 μm. Pixel size, 200 nm. Additional file 7: Figure S3. Microglial processes converge towards a 2Me-ADP-containing pipette in a synchronized way. **A**–**B** Examples of maximal intensity projection in the *z* direction and in time for 6 min of two-photon images in slices where 2Me-ADP (100 μM)-containing pipette have been introduced (at *t* = 0) in control (A) and 48 h after the induction of a *SE* (B). Scale bar, 20 μm. Additional file 8: Figure S4. The synchronization of microglial processes is less evident in the movement induced by laser lesion. Maximal intensity projections in the *z* direction and in time for 135 s (five time frames) of two-photon images in control. Processes arriving from the cell on the left reached the lesion before other processes, and there is no organization in circle as observed when a 2Me-ADP-containing pipette is inserted (see Additional file 7: Figure S3). Additional file 9: Figure 5. Activated microglia lose their spatial segregated distribution. Three-dimensional reconstruction of 2-P image stacks obtained in control (top row) and KA-injected (bottom row) animals. Imaris software was used to reconstruct the morphology of single microglial cells, and to each cell, a different color was associated. Scale grid, 10 μm.

## Abbreviations

2Me-ADP: 2-Methylthioadenosine diphosphate trisodium salt
aCSF: artificial cerebrospinal fluid
KA: kainate
SE: status epilepticus
