# Supplementary figures and images for: Altered morphological dynamics of activated microglia after induction of status epilepticus
Source: J Neuroinflammation. 2015 Nov 4;12:202. doi: 10.1186/s12974-015-0421-6 (PMC4634193; doi:10.1186/s12974-015-0421-6)

**A**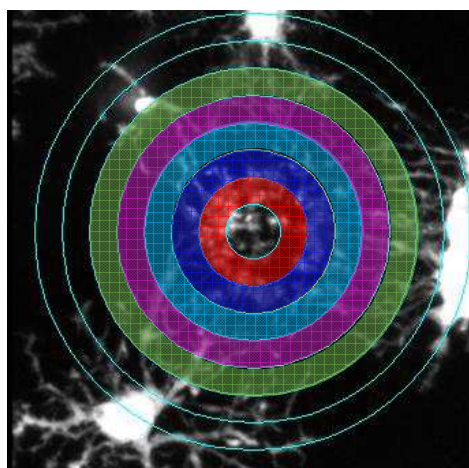**B**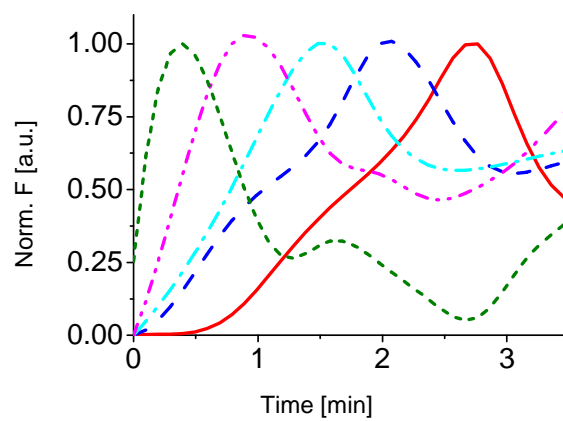

Supplement: Additional file 1: Figure S1. — Method to assess global velocity of microglial processes. A Concentric rings are drawn around the area of interest (laser lesion spot or 2Me-ADP-containing pipette) in the maximal intensity projection (MIP) image. B Global velocity is calculated by observing the fluorescent wave passing through the rings in the MIP video. Each curve represents the evolution of the normalized fluorescence in time in the corresponding ring (matched color in A). [file 12974_2015_421_MOESM1_ESM.pdf]

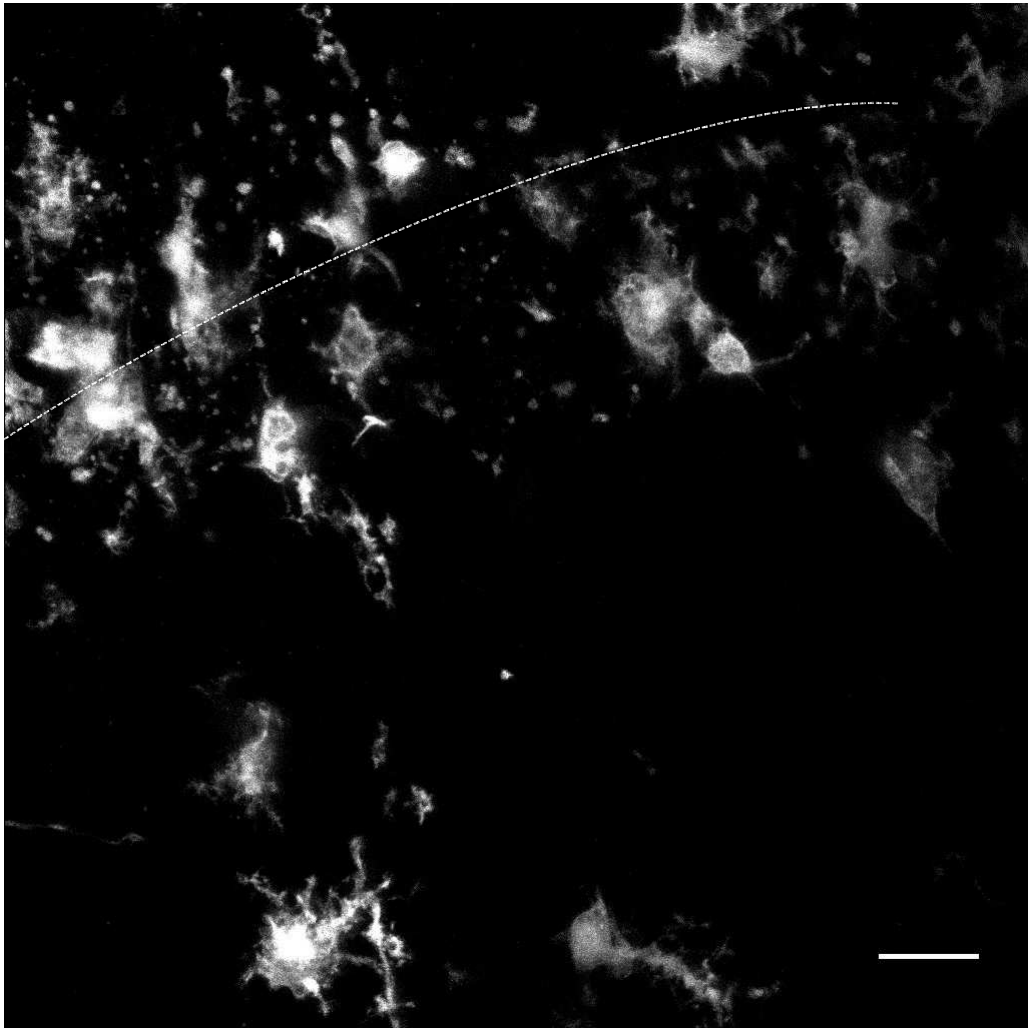

Avignone et al. Supplementary Figure 2

Supplement: Additional file 2: Figure S2. — Example of hyper-activated microglial cells in a slice obtained from a mouse with a long and severe crisis. Maximal intensity projection of two-photon images of the CA1 region in a hippocampal slice obtained from a KA-injected animal, which showed a particularly severe crisis. Microglia almost completely retracted their processes and accumulated around the stratum pyramidale, indicated by the dotted line. Scale bar, 30 μm. [file 12974_2015_421_MOESM2_ESM.pdf]

**A**

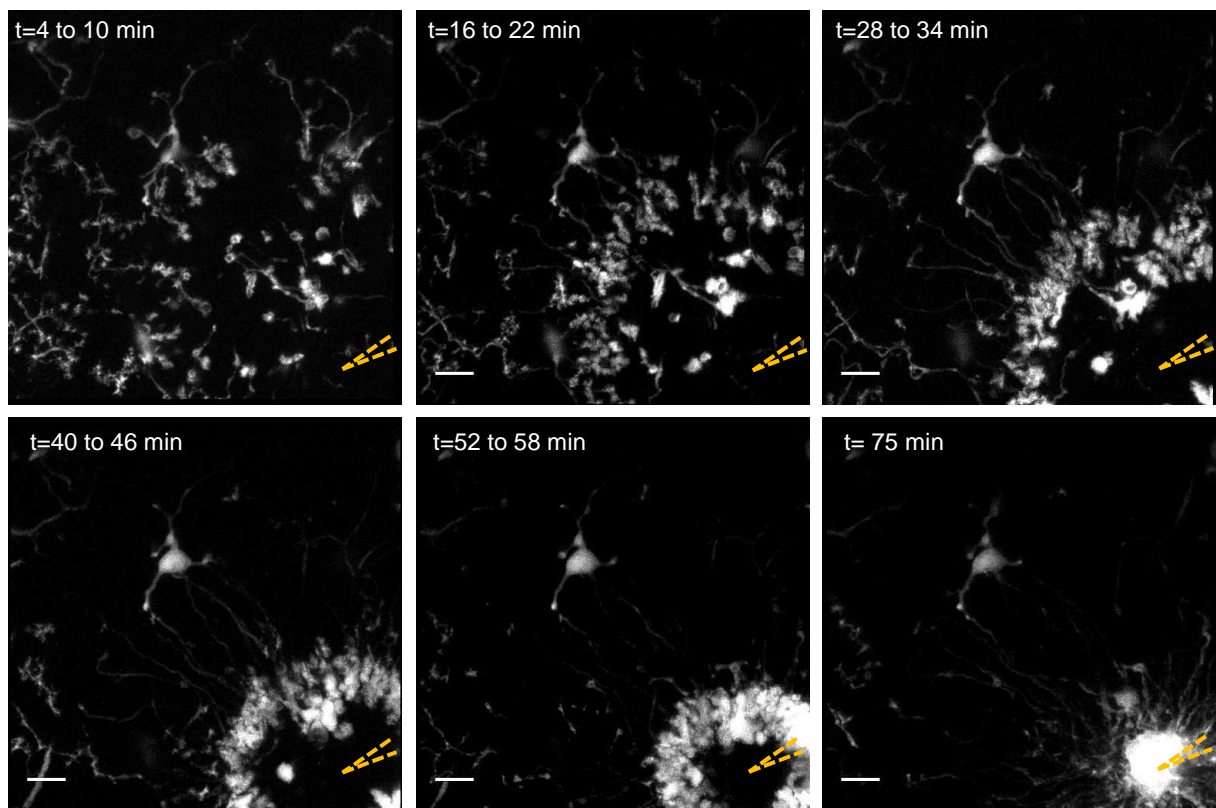

**B**

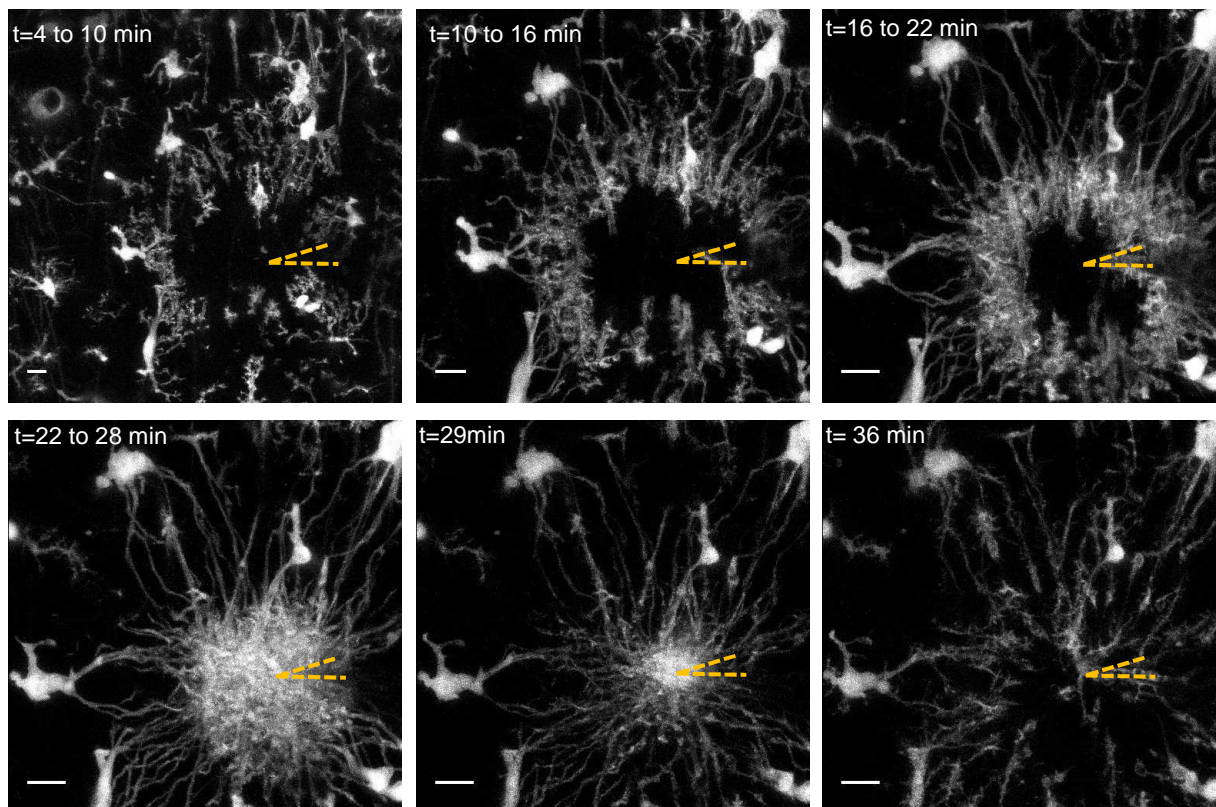

Supplement: Additional file 7: Figure S3. — Microglial processes converge towards a 2Me-ADP-containing pipette in a synchronized way. A–B Examples of maximal intensity projection in the z direction and in time for 6 min of two-photon images in slices where 2Me-ADP (100 μM)-containing pipette have been introduced (at t = 0) in control (A) and 48 h after the induction of a SE (B). Scale bar, 20 μm. [file 12974_2015_421_MOESM7_ESM.pdf]

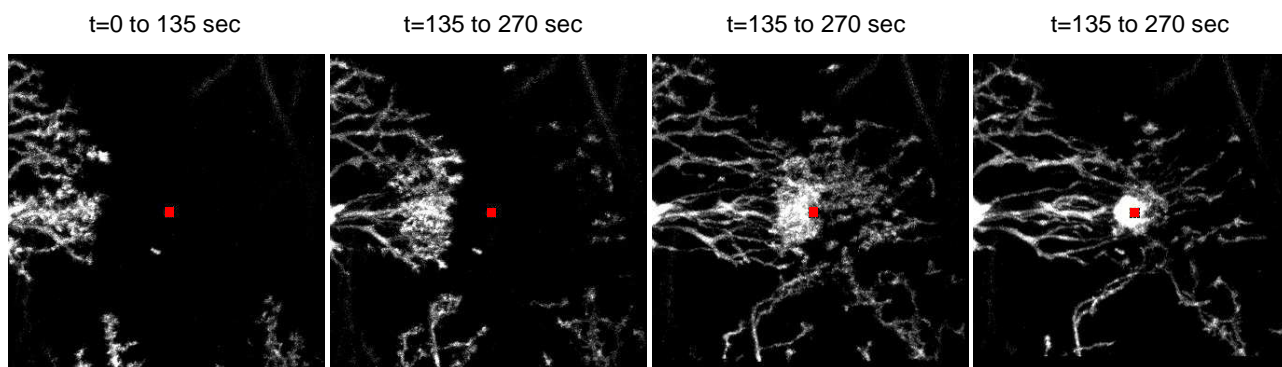

Avignone et al. Supplementary Figure 4

Supplement: Additional file 8: Figure S4. — The synchronization of microglial processes is less evident in the movement induced by laser lesion. Maximal intensity projections in the z direction and in time for 135 s (five time frames) of two-photon images in control. Processes arriving from the cell on the left reached the lesion before other processes, and there is no organization in circle as observed when a 2Me-ADP-containing pipette is inserted (see Additional file 7: Figure S3). [file 12974_2015_421_MOESM8_ESM.pdf]

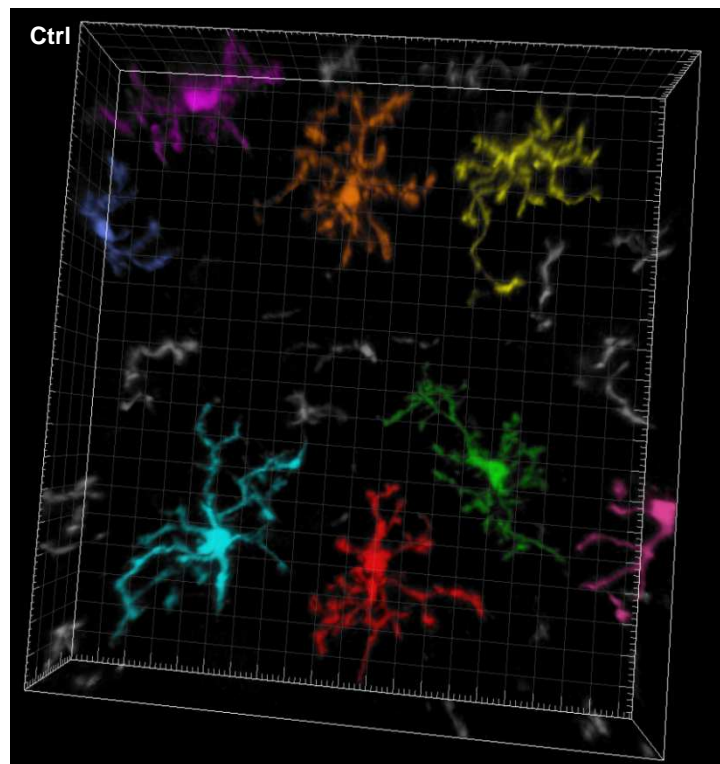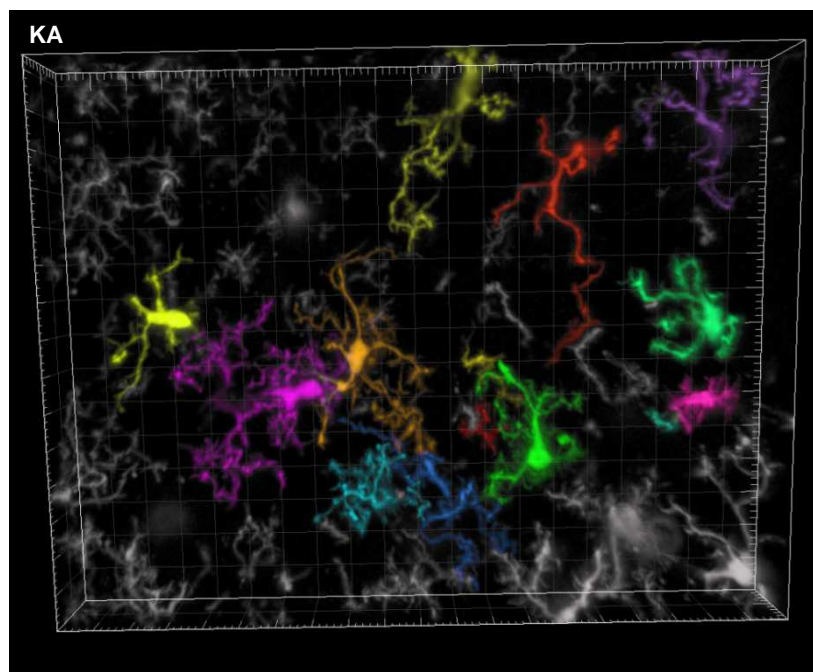

Avignone et al. Supplementary Figure 5

Supplement: Additional file 9: Figure 5. — Activated microglia lose their spatial segregated distribution. Three-dimensional reconstruction of 2-P image stacks obtained in control (top row) and KA-injected (bottom row) animals. Imaris software was used to reconstruct the morphology of single microglial cells, and to each cell, a different color was associated. Scale grid, 10 μm. [file 12974_2015_421_MOESM9_ESM.pdf]
